# Replicability or revision? Further evidence on the psychometric quality of the German version of the epistemic trust, mistrust and credulity questionnaire

**DOI:** 10.1186/s40359-026-04810-0

**Published:** 2026-05-26

**Authors:** Nicola-Hans Schwarzer, Noëlle Behringer, Paula Dees, Stephan Gingelmaier, Melanie Henter, Holger Kirsch, Tillmann Kreuzer, Robert Langnickel, Pierre-Carl Link, Sascha Müller, Agnes Turner, Tobias Nolte

**Affiliations:** 1 Department of Special Education, Heidelberg University of Education, Keplerstr. 87, Heidelberg, 69120 Germany; 2 Department of Social Work and Health Care, Ludwigshafen University of Business and Society, Ludwigshafen, Germany; 3 Department of Emotional and Social Development, Ludwigsburg University of Education, Ludwigsburg, Germany; 4 Department for Special Education, University of Kaiserslautern-Landau, Landau, Germany; 5 University of Applied Sciences, Darmstadt, Germany; 6 Department for Special Education, Freiburg University of Education, Freiburg, Germany; 7 Department for Diversity and Inclusive Education, Luzern University of Education, Luzern, Switzerland; 8 Institute for Educational Support for Behaviour, Social-Emotional, and Psychomotor Development, University of Teacher Education in Special Needs Zürich, Zürich, Switzerland; 9 Department of Psychosomatic Medicine, Rostock University Medical Center, Rostock, Germany; 10 Department of Instructional and School Development, University of Klagenfurt, Klagenfurt, Austria; 11 Anna Freud, London, UK; 12 Research Department of Clinical, Educational and Health Psychology, University College London, London, UK

**Keywords:** Epistemic trust, Epistemic mistrust, Epistemic credulity, Psychometric evaluation, Factor analysis

## Abstract

**Background:**

The Epistemic Trust, Mistrust, and Credulity Questionnaire (ETMCQ) assesses individual differences in epistemic stances. Although widely used, its German translation has not yet undergone an independent psychometric evaluation.

**Method:**

We administered the German ETMCQ to 567 university students and performed confirmatory factor analyses to compare the original 15-item model with several published modifications to further examine psychometric properties of the instrument. We assessed model fit, convergent validity, composite reliability, and internal consistency. Nomological validity was evaluated via latent correlations with attachment insecurity, ineffective mentalizing, emotion regulation, childhood maltreatment, and mental health symptoms.

**Results:**

Neither the 15-item nor the 12-item models achieved acceptable fit. Across all models tested, average variance extracted values remained below 0.50, and composite reliabilities failed to reach Jöreskog’s rho *ρ* ≥ 0.70 for any of the three subscales. Internal consistency was marginally acceptable for the trust, mistrust, and credulity subscales. Nomological analyses revealed theory-consistent associations for mistrust and credulity but revealed weaker, divergent patterns for trust.

**Conclusions:**

These findings highlight psychometric limitations in the current German version of the ETMCQ in this specific population. Before broader use, we recommend comprehensive item revision, multimethod validation, and cross-language invariance testing.

**Supplementary Information:**

The online version contains supplementary material available at 10.1186/s40359-026-04810-0.

## Introduction

Drawing on its origins in different disciplines, such as philosophy [1], sociology [2], and epistemology [3], Campbell and colleagues [4] refer to the underlying expectations of the information recipient in human communication processes as an ‘epistemic stance’. According to Campbell and colleagues [4], this stance can further be described by the three overlapping dimensions of epistemic trust, epistemic mistrust, and epistemic credulity, which individuals may adopt toward information senders.

Epistemic trust is grounded in the reliable experience of being acknowledged as an agentive, unique individual with an independent mental experience during social interactions [5] – for example, in attachment relationships classified as secure, where the child consistently experiences being mentalized (i.e., being perceived in their mental states) by the caregiver [6]. Such relational experiences foster the development of epistemic trust, enabling the individual to regard the information conveyed in social communication as personally relevant, trustworthy, and generalizable [7, 8]. This in turn facilitates an efficient exchange of socially and culturally pertinent information [5, 9]. Thus, epistemic trust is regarded as a central prerequisite for learning from others in social contexts [10, 11, 12] – even when previously unknown information is perceived as opaque [13]. It is deemed essential for effective interpersonal functioning, mentalizing, and ultimately represents a key factor influencing mental health by underpinning resilience against psychopathology [14–17].

In contrast, Fonagy and colleagues [10, 14, 18] postulate that depriving, aversive, or threatening developmental circumstances impede the development of epistemic trust and instead maladaptively amplify an inherent epistemic hypervigilance, thereby fostering epistemic mistrust or epistemic credulity. More precisely, epistemic vigilance refers to an adaptive, evolutionarily grounded mechanism that enables individuals to critically evaluate the reliability and relevance of socially conveyed information [7]. In doing so, it serves as a protective filter that helps balance openness to communication with caution against deception or misinformation [10]. However, when this vigilance becomes overgeneralized and rigid, it may culminate in either epistemic mistrust or epistemic credulity, both of which represent maladaptive manifestations of epistemic processing that are considered core features of various mental disorders [5, 11].

Epistemic mistrust is characterized by a persistently rigid state of psychological withdrawal, coupled with a deeply rooted conviction that other people are untrustworthy and that socially transmitted information – irrespective of whether it pertains to cognitive or emotional-social knowledge – is perceived as irrelevant, ill-intended, or inconsequential [10]. Notably, these dysfunctional developments are understood as adaptive responses that, while functional in coping with adverse developmental circumstances and preserving basic functioning, significantly hinder the updating of emotional-social and cognitive knowledge based on social information, ultimately leading to an interpersonally inaccessible and socially isolated epistemic stance [19].

Besides epistemic trust and epistemic mistrust, epistemic credulity is described by Campbell and colleagues [4] as a third epistemic stance, defined by a compromised ability to discriminate between pieces of information and characterized by an excessive and inappropriate trust in others. Consequently, socially conveyed information is accepted uncritically, rendering individuals more susceptible to manipulation, exploitation, and abuse.

Within this conceptual framework, epistemic stances are assumed to shape how individuals interpret and respond to interpersonal experiences and are therefore considered central to human psychosocial development [11, 12]. Specifically, trustful epistemic orientations – formed through sensitive and well-attuned affective relationships [5, 6] – enable individuals to mentalize effectively and to regulate emotions in an adaptive manner. In contrast, misattuned or hostile early relational experiences are thought to foster epistemic mistrust and credulity, which are, in turn, associated with maladaptive developmental trajectories characterized by emotional dysregulation, attachment insecurity, and increased vulnerability to psychopathology [10, 14, 18].

### Overview of the current state of research

In recent years, a significant increase in studies has been documented that examine the framework of the epistemic stance in relation to human psychosocial development. Consistent with the theoretical framework presented above, current findings indicate that disturbances in epistemic functioning are associated with traumatic biographical experiences – such as abuse and neglect – in clinical [20], mixed [21], and population-representative samples [22].

In addition, empirical evidence supports the hypothesized relationships between epistemic disturbances and psychopathology. For instance, epistemic mistrust has been linked to interpersonal problems [23], psychological maladjustment [24], impaired personality functioning [25], attachment insecurity [26], and increased symptom severity [27]. Similarly, studies indicate that epistemic credulity is associated with adverse psychological outcomes, including symptoms and loneliness [28], difficulties in emotion regulation [29], and engagement in conspiracy beliefs [30, 31].

By contrast, the association between psychopathology and epistemic trust is less consistent. Some studies report negative associations [32, 33], whereas others find these relationships to be inconsistent [4] or non-significant [22, 27]. This variability has led some authors to question the incremental validity of epistemic trust [34].

Building on these findings, conceptual research has increasingly emphasized the role of the epistemic stance in the effectiveness of psychological interventions [16, 17]. Evidence from several studies indicates that epistemic stances can be fostered through psychosocial interventions [35–37], and that improvements in epistemic trust are associated with reductions in symptoms and impairments [35, 36].

### Assessment of epistemic trust, mistrust, and credulity

Given the promising conceptual framework, instruments used to assess epistemic trust, epistemic mistrust, and epistemic credulity that exhibit high psychometric quality are a crucial prerequisite for obtaining robust findings. In addition to performance-based measures [38], recently published self-report instruments [39] are particularly well-suited for the economical assessment of epistemic stances in large samples.

Another questionnaire that is available in various languages and has increasingly been used in scientific studies is the Epistemic Trust, Mistrust, and Credulity Questionnaire (ETMCQ) [4]. Initially developed in English, the ETMCQ originally consisted of 18 Likert-scale items (1 = strongly disagree, 7 = strongly agree), with six items loading on each of the three factors: epistemic trust (e.g., “If I don’t know what to do in a particular situation, my first instinct is to ask someone whose opinion I value”), epistemic mistrust (e.g., “If you put too much faith in what people tell you, you are likely to get hurt”), and epistemic credulity (e.g., “When I speak to different people, I find myself easily persuaded even if it is not what I believed before”).

Based on the results of factor analyses from two independent samples, Campbell and colleagues [4] report that the original 18-item version of the questionnaire demonstrated inadequate fit indices, prompting a data-driven modification of the original scale. This modification resulted in a 15-item version with acceptable psychometric fit (Sample 1: Root Mean Square Error of Approximation (RMSEA) = 0.07 [0.06 – 0.08]; Comparative Fit Index (CFI) = 0.95; Tucker-Lewis Index (TLI) = 0.94; Standardized Root Mean Square Residual (SRMR) = 0.05; Sample 2: RMSEA = 0.08 [0.07 – 0.08], CFI = 0.94; TLI = 0.92; SRMR = 0.05). Specifically, during the examination of the factorial structure, three of the original items were excluded due to weak factor loadings (*λ* < 0.40), and five error correlations were allowed due to similar content and wording. The internal consistencies of the three scales ranged from *α* = 0.65 to *α* = 0.81, which the authors considered acceptable [4].

In the process of translating the questionnaire into other languages, further psychometric evaluations were conducted that used the 15-item version recommended by Campbell and colleagues [4] as a basis. However, these studies revealed that although the three-factor structure was replicated roughly, additional modifications were required to achieve acceptable fit indices. Consequently, alternative versions of the questionnaire have been proposed that consist of either 14 items [26, 29] or 12 items [28], with up to four correlated error terms between individual items permitted.

Of particular interest for the present study are recent findings by Weiland and colleagues [20] pertaining to the German version of the ETMCQ. Their results revealed that, while the three-factor structure of the questionnaire was broadly supported, data-driven modifications were still necessary to achieve an acceptable model fit. Specifically, Weiland and colleagues [20] recommend removing three items due to insufficient factor loadings (*λ* < 0.50) and permitting two correlated error terms, which resulted in an acceptable model fit (*χ²*(49, 584) = 119.91, *p* < .001; RMSEA = 0.05 [0.04 – 0.07]; CFI = 0.96; TLI = 0.94; SRMR = 0.04).

For the modified 12-item version, Weiland and colleagues [20] report internal consistency coefficients within the acceptable range (epistemic trust *ω* = 0.82; epistemic mistrust *ω* = 0.64; epistemic credulity *ω* = 0.87). A small negative latent correlation was observed between epistemic trust and epistemic mistrust (*r* = –.12, *p* < .05), while no significant association was found between epistemic trust and epistemic credulity (*r* = .08, *p* > .05). In contrast, a substantial latent correlation emerged between epistemic mistrust and epistemic credulity (*r* = .82, *p* < .001). Unexpectedly, the German version of the ETMCQ was unable to differentiate between clinical and non-clinical participants. Consistent with the theoretical framework, Weiland et al. [20] also report significant associations between epistemic mistrust, epistemic credulity, and a history of maltreatment, impaired personality functioning, and ineffective mentalizing. In contrast, the hypothesized relationships between epistemic trust, mentalizing, a history of maltreatment, and personality functioning were only partially confirmed.

### The current study

Given the increasing use of the German version of the ETMCQ in empirical studies [22, 25, 35, 36], a comprehensive psychometric evaluation of the instrument as well as its replication across different populations is essential. Recent modifications of the ETMCQ have produced divergent item configurations and suggest that the measurement properties of the instrument may vary across languages. To date, however, the German version has been psychometrically evaluated only by Weiland et al. [20]. Therefore, we prioritized an independent replication of the 12-item model proposed by Weiland et al. [20] while situating our findings in the broader context of alternative solutions reported for other language versions. In this respect, the present study was not intended merely to re-examine a previously proposed German model, but also to evaluate whether the currently available German adaptation provides a sufficiently robust operationalization of epistemic trust, mistrust, and credulity in an independent sample – an issue that is particularly relevant given the increasing use of the German ETMCQ [22, 25, 35, 36]. More broadly, the study addresses a methodological question relevant to cross-linguistic questionnaire adaptation, namely whether repeated item-level modifications across language versions may indicate problems in preserving the intended construct representation of the original instrument, as proposed by Campbell et al. [4].

The aim of the present study was, firstly, to replicate the three-factor structure of the German version of the ETMCQ as specified by Weiland et al. [20] using confirmatory factor analysis. Furthermore, the study aimed to examine the discriminant validity of the items, determine the composite reliabilities, and assess the internal consistency of the three factors. Additionally, to further substantiate the validity of the German version of the ETMCQ proposed by Weiland et al. [20], the study investigated the relationships between epistemic trust, epistemic mistrust, and epistemic credulity and the theoretically key constructs of a history of maltreatment and neglect, ineffective mentalizing, attachment insecurity, emotion regulation, and mental health. Thus, beyond testing the fit of one specific model, the present study also sought to clarify whether the current German ETMCQ can be interpreted as a psychometrically defensible measure of the target constructs in its presently available form. Based on this framework, the following hypotheses were formulated:

Hypothesis H1: It is expected that a modified short version of the ETMCQ suggested by Weiland et al. [20] will better capture the three-factor structure of the ETMCQ than the original 15-item version.

Hypothesis H2: Satisfactory coefficients for both composite reliability and internal consistency of all three factors are expected for this model.

Hypothesis H3: In line with theoretical expectations, significant statistical relationships are anticipated between epistemic stances – when operationalized as per Weiland et al. [20] – and attachment insecurity, ineffective mentalizing, experiences of abuse and neglect, emotion regulation, and mental health.

## Materials and methods

### Research design and participants

The data for this cross-sectional study were collected as part of a research project aiming to validate a novel instrument for assessing mentalizing capacities in teachers and educators. Data collection was conducted via the online survey platform SoSci Survey. Participants were recruited through university courses, institutional email lists, and bulletin board announcements at several German universities. Participation was voluntary, fully anonymized, and could be discontinued at any time without negative consequences.

Eligibility criteria included enrollment in a pedagogical degree program (e.g., teacher education or educational science), a minimum age of 18 years, and provision of informed consent for the use of data. To reduce potential order effects, the presentation of the questionnaires was randomized across participants. Participants who failed attention checks were excluded from the analyses. Ethical approval was obtained from the Ethics Committee of Ludwigsburg University of Education (III-Sopaed_NiSc-0018). Prior to participation, all individuals were informed about the study’s objectives and provided written consent. All procedures were conducted in accordance with the principles of the Declaration of Helsinki [40].

The final sample consisted of 567 participants, primarily young adults (411 female, 81 male, 3 non-binary, 72 undisclosed), recruited from educational degree programs across several German universities. The average age was 27.22 years (*SD* = 8.52; range: 19–60 years). Analyses revealed a statistically significant age difference between female and male participants, albeit with a small effect size (*F*(2, 493) = 6.23, *p* ≤ .01, *η²* = 0.03), indicating that male and non-binary participants were slightly older than female participants on average.

### Measures

In addition to the ETMCQ [4] for assessing epistemic trust, epistemic mistrust, and epistemic credulity, the following measures were used in the current study:

#### Childhood maltreatment

A history of maltreatment was assessed with the Childhood Trauma Questionnaire (CTQ) [41], using the German version [42]. The CTQ is a reliable and valid self-report instrument comprising 28 items that measure various aspects of childhood abuse, including physical abuse, sexual abuse, emotional abuse, as well as physical and emotional neglect. Each subscale consists of five items, and respondents rate their experiences on a five-point Likert scale (1 = never true, 5 = very often true). In the current study, higher scores indicate a history of severe childhood maltreatment. The internal consistencies of the subscales are considered acceptable given the scale length, with values ranging from *ω* = 0.53 to *ω* = 0.86. In the present study, a confirmatory factor analysis using robust maximum-likelihood estimation largely confirmed the internal structure of the latent maltreatment factor based on the five CTQ subscales. Model fit indices suggested an adequate overall fit (*χ²*(5, 567) = 27.09, *p* ≤ .001; RMSEA = 0.13 [0.08 – 0.18]; CFI = 0.94; TLI = 0.89; SRMR = 0.04).

#### Ineffective mentalizing

Ineffective mentalizing was assessed using the short German version of the Reflective Functioning Questionnaire (RFQ) [43], which measures individual tendencies to consider mental states as relevant for understanding one’s own and others’ behavior. The RFQ is considered a reliable and valid self-report instrument that is both economical and suitable for large samples. It consists of eight statements rated on a seven-point Likert scale (1 = completely disagree to 7 = completely agree). Based on three large clinical and non-clinical samples from Germany and the US, Müller et al. [44] recommended using the RFQ as a unidimensional scale comprising only six items that exclusively measure uncertainty in using mental states as reliable information (e.g., “I don’t always know why I do what I do”). The internal consistency of the scale was acceptable (*ω* = 0.69). In the present sample, a confirmatory factor analysis with robust maximum-likelihood estimation confirmed the expected latent structure of this factor. All model fit indices were within the acceptable range (*χ²*(9, 567) = 24.47, *p* = .004; RMSEA = 0.06 [0.03 – 0.09]; CFI = 0.97; TLI = 0.95; SRMR = 0.03).

#### Attachment insecurity

Attachment insecurity was assessed using the German version of the Experiences in Close Relationships – Revised Questionnaire in its short form (ECR-RD8) [45]. The ECR-RD8 is a self-report measure that requires participants to respond to eight statements on a seven-point Likert scale (1 = strongly disagree, 7 = strongly agree). Specifically, the ECR-RD8 comprises two subscales: one measuring attachment-related anxiety (e.g., “I rarely worry about my partner leaving me”) and another assessing attachment-related avoidance (e.g., “It is not difficult for me to get close to my partner” [reverse coded]). High scores on both subscales indicate stronger expressions of attachment-related anxiety or avoidance. Ehrenthal et al. [45] reported acceptable psychometric properties for the instrument, which were confirmed in the current study **(**attachment-related anxiety: *ω* = 0.75; attachment-related avoidance: *ω* = 0.78). In the present study, a confirmatory factor analysis using robust maximum-likelihood estimation revealed acceptable fit indices (*χ²*(19, 567) = 93.97, *p* < .001; RMSEA = 0.09 [0.07 – 0.10]; CFI = 0.95; TLI = 0.92; SRMR = 0.05).

#### Emotion regulation

Emotion regulation was assessed using the German version of the Emotion Regulation Questionnaire (ERQ) [46]. The ERQ is considered a reliable instrument with excellent psychometric properties [47]. It measures two fundamental emotion regulation processes – suppression and reappraisal – through ten items rated on a seven-point Likert scale (1 = completely disagree, 7 = completely agree). Higher scores on the reappraisal subscale indicate more adaptive emotion regulation (e.g., “When I’m faced with stressful situations, I make myself think about it in a way that helps me to stay calm”). In contrast, higher scores on the suppression subscale reflect a more maladaptive approach to regulating emotional states (e.g., “When I am feeling negative emotions, I make sure not to express them”). The internal consistency of both subscales was good (reappraisal: *ω* = 0.87; suppression: *ω* = 0.75). A confirmatory factor analysis using robust maximum-likelihood estimation revealed acceptable fit indices for the measurement model in the present sample (*χ²*(34, 567) = 109.33, *p* < .001; RMSEA = 0.07 [0.06 – 0.08]; CFI = 0.95; TLI = 0.93; SRMR = 0.05).

#### Mental health

A brief version of the Symptom Checklist (SCL) [48] was used to assess mental health. The SCL-9 comprises nine Likert-scale items (1 = not at all, 5 = very much; e.g., “How much have you suffered from feelings of powerlessness in the past 7 days?”), serving as a screening tool with excellent psychometric properties, as confirmed in various studies [49]. High scores indicate a higher level of symptom severity. The internal consistency of the scale was good (*ω* = 0.82). In the present sample, a confirmatory factor analysis using robust maximum-likelihood estimation on the nine items of the SCL-9 confirmed the latent structure of the construct, with all model fit indices falling within acceptable ranges (*χ²*(27, 567) = 95.58, *p* < .001; RMSEA = 0.08 [0.06 – 0.09]; CFI = 0.93; TLI = 0.91; SRMR = 0.04).

### Data analytic plan

Analyses were conducted using IBM SPSS (Version 29) and R (Version 4.5.1) with the packages haven [50], lavaan [51], and dplyr [52]. In the first step, cases with completely unprocessed data were excluded (*n* = 0). Second, cases that failed to answer both attention-check items correctly were excluded (*n* = 52). Because all items were set as mandatory in the online survey, no missing data occurred.

To assess the factorial structure of the ETMCQ, several confirmatory factor analyses (CFAs) were conducted. Model fit was evaluated using the fit indices recommended by Hu and Bentler [53]: the *χ²* statistic, TLI, RMSEA, CFI, and SRMR. In addition, the Akaike Information Criterion (AIC) and Bayesian Information Criterion (BIC) were considered, with lower values indicating superior model fit. For comparisons between nested models, a robust chi-square difference test was additionally used. Because the 12-item model proposed by Weiland et al. [20] differed from the two 15-item models in the number of retained indicators, not all compared models were nested. Accordingly, approximate fit indices served as the primary basis for evaluating all three model specifications, whereas chi-square difference testing was used specifically for the comparison between the two nested 15-item models. Thresholds for good fit were a non-significant χ² statistic, RMSEA ≤ 0.06, TLI ≥ 0.95, CFI ≥ 0.95, and SRMR ≤ 0.06 [53].

Three versions of the measurement model were compared: (1) the unmodified 15-item version, (2) the 15-item solution proposed by Campbell et al. [4], and (3) the 12-item German adaptation by Weiland et al. [20]. All CFAs used robust maximum-likelihood estimation to account for potential deviations from multivariate normality [51]. The sample size (*N* = 567) meets established recommendations for confirmatory factor analyses, which typically suggest at least five to ten participants per estimated parameter or approximately 400 participants to ensure stable model estimation [54]. Accordingly, the study can be considered adequately powered for the conducted analyses.

Reliability for the three ETMCQ scales was estimated using McDonald’s omega (*ω*). For comparability with previous research, Cronbach’s alpha (*α*) is also reported. Convergent validity was evaluated via the average variance extracted (AVE), with values ≥ 0.50 considered adequate [55]. Composite reliability was further assessed using the Jöreskog rho coefficient (acceptable threshold: *ρ* ≥ 0.70) [56]. While McDonald’s omega (ω) estimates the proportion of true-score variance in observed scores based on classical test theory, composite reliability (Jöreskog’s *ρ*) provides a model-based SEM estimate that reflects the reliability of latent constructs derived from factor loadings and error variances [56]. Nomological validity of the ETMCQ scales – when assessed as per Weiland et al. [20] – was examined by estimating latent correlations between the three ETMCQ scales and the external criteria of a history of abuse and neglect, attachment insecurity, mentalizing, emotion regulation, and mental health, using robust maximum-likelihood estimation to account for potential deviations from multivariate normality.

## Results

The fit indices from the confirmatory factor analyses (CFAs) are presented in Table 1. The CFA of the unmodified 15-item model demonstrated poor fit. Similarly, the 15-item solution proposed by Campbell et al. [4] failed to meet the cut-off criteria, yielding unsatisfactory fit and inadequately representing the data. However, a robust chi-square difference test indicated that the 15-item model proposed by Campbell et al. [4] fit the data significantly better than the unmodified 15-item model (Δχ²(4) = 137.06, *p* < .001). Although the 12-item variant from Weiland et al. [20] approximated the observed data more closely, its fit indices remained below the predefined thresholds, indicating persistent structural misfit. Moreover, inspection of modification indices consistently revealed substantial cross-loadings, suggesting that several items did not clearly align with their intended factors, as previously noted by Weiland et al. [20]. An overview of the item composition of all three ETMCQ models, standardized path diagrams for the three CFA models, and additional item-level information, including descriptive statistics, inter-item correlations, and item-level diagnostics for the German ETMCQ, are provided in the Electronic Supplementary Material (ESM Materials 1 and 2).

**Table 1:** Fit indices of the confirmatory factor analyses

Model	Items	df	χ^2^	CFI	TLI	RMSEA [90% CI]	SRMR	AIC	BIC
unmodified	15	87	314.44***	0.81	0.77	0.07 [0.06, 0.08]	0.07	28787.34	28930.57
Campbell et al. (2021) [4]	15	83	206.31***	0.90	0.87	0.05 [0.04, 0.06]	0.06	28681.04	28841.64
Weiland et al. (2024) [20]	12	49	115.28***	0.93	0.91	0.05 [0.04, 0.06]	0.05	23049.71	23175.58

*N* =567

*df* degrees of Freedom, *CFI* Comparative Fit Index, *TLI* Tucker–Lewis Index, *RMSEA* Root Mean Square Error of Approximation, *CI* confidence interval, *SRMR* Standardized Root Mean Square Residual, *AIC* Akaike Information Criterion, *BIC* Bayesian Information Criterion

*** p < .001, ** *p* < .01, * *p* < .05

The standardized factor loadings for all items across the three CFA model variants are presented in Table 2. Additionally, we report reliability coefficients for each scale (Cronbach’s *α*, McDonald’s *ω*), the average variance extracted (AVE), and composite reliabilities (Jöreskog’s rho). Despite the limited number of items, internal consistency was generally acceptable across all models, except for the mistrust factor, which consistently demonstrated weak internal consistency. At the same time, all three models failed the Fornell–Larcker criterion, with AVE values below 0.50, indicating that less than half of the variance in each factor was explained by its items. Likewise, composite reliabilities (*ρ*) for all three factors fell below the recommended threshold of 0.70 in every model tested, with the sole exception of the credulity factor in the original 15-item version, which achieved *ρ* ≥ 0.70.

**Table 2:** Results of the confirmatory factor analyses

	unmodified			Campbell et al. (2021)			Weiland et al. (2024)		
	trust	mistrust	credulity	trust	mistrust	credulity	trust	mistrust	credulity
ETMCQ 1	0.68			0.70			0.69		
ETMCQ 2	0.27			0.27			0.28		
ETMCQ 7	0.50			0.48			0.47		
ETMCQ 8	0.32			0.29			0.28		
ETMCQ 13	0.65			0.65			0.66		
ETMCQ 3		0.40			0.40				
ETMCQ 4		0.64			0.63			0.69	
ETMCQ 9		0.39			0.39			0.35	
ETMCQ 10		0.33			0.33			0.27	
ETMCQ 14		0.49			0.50				
ETMCQ 5			0.65			0.42			0.43
ETMCQ 6			0.37			0.21			
ETMCQ 11			0.58			0.73			0.73
ETMCQ 12			0.70			0.48			0.49
ETMCQ 15			0.55			0.70			0.69
trust	–			–			–		
mistrust	− 0.43***	–		− 0.43***	–		− 0.29***	–	
credulity	0.17**	0.52***	–	0.11	0.57***	–	0.11	0.71***	–
AVE	0.26	0.21	0.34	0.26	0.21	0.29	0.26	0.22	0.36
α	0.61	0.56	0.70	0.61	0.56	0.70	0.61	0.42	0.71
ω	0.62	0.56	0.69	0.62	0.56	0.69	0.62	0.42	0.68
ρ	0.61	0.56	0.71	0.60	0.56	0.64	0.60	0.42	0.68

*N =567*

*AVE* average variance extracted, *α* Cronbach’s alpha, *ω* McDonald’s omega coefficient, *ρ* Jöreskog’s rho (composite reliability coefficient)

*** *p* < .001, ** *p* < .01, * *p* < .05

Because none of the tested CFA models showed fully acceptable fit, we conducted a post hoc exploratory factor analysis (EFA) as a follow-up analysis to better understand the remaining structural misfit and the instability of item-to-factor assignments. The EFA used maximum-likelihood extraction and direct oblimin rotation to allow for correlated factors [56]. The number of factors was fixed to three, in accordance with the theoretical model [4]. The rotated pattern matrix is provided in Table 3. The sample was adequate for factor analysis, as indicated by the Kaiser–Meyer–Olkin (KMO) value of 0.74 and Bartlett’s test of sphericity (*χ²*(105) = 1400.23, *p* < .001) [56]. Six items (Items 2, 3, 8, 9, 10, and 14) did not meet the loading threshold of *λ* ≥ 0.40 and showed ambiguous loading patterns. Importantly, two items originally assigned to the credulity factor (Items 11 and 15) and one item originally assigned to the mistrust factor (Item 4) loaded together on a single latent factor, suggesting overlap in item content. Moreover, Item 6 demonstrated sufficient loading in the present sample to warrant retention, contradicting Weiland et al.’s [20] recommendation to exclude this item.

**Table 3:** Results of the exploratory factor analysis

	factor 1	factor 2	factor 3
ETMCQ_1	0.153	0.663	− 0.178
ETMCQ_2	0.089	0.263	0.020
ETMCQ_3	− 0.019	− 0.369	0.275
ETMCQ_4	0.309	− 0.248	**0.565**
ETMCQ_5	**0.753**	0.142	0.275
ETMCQ_6	**0.421**	0.145	0.108
ETMCQ_7	0.069	**0.515**	− 0.131
ETMCQ_8	0.073	0.326	− 0.177
ETMCQ_9	0.157	− 0.109	0.360
ETMCQ_10	0.034	− 0.154	0.270
ETMCQ_11	0.381	0.092	**0.639**
ETMCQ_12	**0.726**	0.058	0.379
ETMCQ_13	0.232	**0.613**	− 0.110
ETMCQ_14	0.038	− 0.343	0.391
ETMCQ_15	0.365	0.074	**0.622**

*N* = 567

Bold coefficients indicate the factor on which each item showed its highest loading

In the final step, we evaluated the nomological validity of the Weiland et al. [20] measurement model by estimating latent correlations between its three ETMCQ subscales and key constructs (see Table 4). Epistemic mistrust exhibited strong positive associations with a history of abuse and neglect (*r* = .42, *p* < .001), ineffective mentalizing (*r* = .52, *p* < .001), and poorer mental health (*r* = .64, *p* < .001), and was negatively correlated with reappraisal (*r* = –.34, *p* < .001). Epistemic credulity showed positive relations with ineffective mentalizing (*r* = .50, *p* < .001), poorer mental health (*r* = .57, *p* < .001), and childhood maltreatment (*r* = .34, *p* < .001), along with a weak negative association with reappraisal (*r* = –.19, *p* < .01). Both mistrust and credulity were positively associated with anxious attachment (*r* = .51; *r* = .44, both *p* < .001). However, only mistrust was significantly related to avoidant attachment (*r* = .31, *p* < .001), whereas credulity was not (*r* = .08, *p* > .05). Differences also emerged for suppression: mistrust correlated positively with suppression (*r* = .35, *p* < .001), but credulity did not (*r* = .10, *p* > .05).

**Table 4:** Latent intercorrelations between the variables (estimation: robust maximum likelihood)

	1	2	3	4	5	6	7	8	9	10
1 ETMCQ_tr	–									
2 ETMCQ_mistr	− 0.28***	–								
3 ETMCQ_cred	0.11	0.65***	–							
4 CTQ	− 0.16*	0.42***	0.34***	–						
5 RFQ	− 0.04	0.52***	0.50***	0.21**	–					
6 ECR_anx	0.03	0.51***	0.44***	0.27***	0.33***	–				
7 ECR_avoi	− 0.17**	0.31***	0.08	0.16**	0.15**	0.42***	–			
8 ERQ_reap	0.22***	− 0.34***	− 0.19**	− 0.12*	− 0.22***	− 0.19***	0.08	–		
9 ERQ_supp	− 0.45***	0.35***	0.10	0.12*	0.25***	0.15**	0.38***	− 0.06	–	
10 SCL	0.03	0.64***	0.57***	0.38***	0.54***	0.52***	0.23***	− 0.34***	0.18**	–

*N = 567*

*ETMCQ_tr* Epistemic Trust, Mistrust, and Credulity Questionnaire trust subscale, *ETMCQ_mistr* Epistemic Trust, Mistrust, and Credulity Questionnaire mistrust subscale, *ETMCQ_cred* Epistemic Trust, Mistrust, and Credulity Questionnaire credulity subscale, *CTQ* Childhood Trauma Questionnaire, *RFQ* Reflective Functioning Questionnaire, *ECR_anx* Experiences in Close Relationships- Questionnaire anxiety subscale, *ECR_avoi* Experiences in Close Relationships- Questionnaire avoidance subscale, *ERQ_reap* Emotion Regulation Questionnaire reappraisal subscale, *ERQ_supp* Emotion Regulation Questionnaire suppression subscale, *SCL* Symptom Checklist

*** *p* ≤ .001, ** *p* ≤ .01, * *p* ≤ .05

By contrast, epistemic trust displayed a divergent profile: it was unrelated to ineffective mentalizing (*r* = –.06, *p* > .05) and mental health symptoms (*r* = .03, *p* > .05), but showed small significant negative correlations with avoidant attachment (*r* = –.17, *p* < .01), a history of abuse and neglect (*r* = –.16, *p* ≤ .05), and with emotion regulation – positively with reappraisal (*r* = .22, *p* < .001) and negatively with suppression (*r* = –.45, *p* < .001). Taken together, these findings suggest that epistemic mistrust and credulity maintain robust nomological links to adversity and maladaptive processes, whereas epistemic trust demonstrates a distinct, more adaptive profile, albeit with comparatively weak associations, underscoring its unique role in psychosocial functioning. 

## Discussion

The present study aimed to further evaluate the psychometric properties of the German version of the Epistemic Trust, Mistrust, and Credulity Questionnaire (ETMCQ) via confirmatory factor analyses, reliability assessments, and tests of nomological validity. Beyond the replication of a previously proposed German model, the study also addressed the broader methodological question of whether the currently available German adaptation provides a sufficiently robust operationalization of epistemic trust, mistrust, and credulity in an independent sample, and what repeated item-level modifications across language versions may imply for construct representation in cross-linguistic adaptation.

Hypothesis 1 – which posited that the 12-item model of Weiland et al. [20] would fit the data better than the original 15-item version – was supported. The 12-item adaptation yielded superior fit relative to both the unmodified 15-item model and the 15-item solution of Campbell et al. [4]. However, in our sample the Weiland model’s fit indices remained below optimal thresholds, raising concerns about the questionnaire’s intended factorial structure and item-to-factor assignments. The exploratory factor analysis further underscored these shortcomings, illustrating psychometric and theoretical inconsistencies. Specifically, six items failed to meet the loading criterion and showed ambiguous factor associations, and two credulity items together with one mistrust item loaded on a shared factor, thereby blurring the theoretical distinction between epistemic mistrust and credulity. Taken together, these results indicate that the latent structure of the current German ETMCQ is not sufficiently stable, and we therefore interpret the exploratory three-factor solution not as an improved alternative, but as further evidence for the conceptual instability of the scale and the need for systematic revision and item redevelopment.

Hypothesis 2 – which predicted that the model of Weiland et al. [20] would yield satisfactory composite reliability and internal consistency across all three subscales – must be rejected. Considering the confirmatory factor analyses, both the original 15-item and the 12‐item Weiland models did not meet convergent‐validity (AVE < 0.50) or composite‐reliability (Jöreskog’s *ρ* < 0.70) criteria for any factor. Although traditional reliability indices were marginally acceptable, they consistently fell below the conventional 0.70 threshold, underscoring persistent psychometric limitations across all assessed configurations.

Hypothesis 3 – which predicted significant associations between epistemic stances and attachment insecurity, ineffective mentalizing, experiences of abuse and neglect, emotion regulation, and mental health – received cautious support. Using the 12-item measurement model proposed by Weiland et al. [20], nomological validity analyses revealed a pattern of associations that was largely consistent with theoretical expectations and previous empirical findings, particularly for epistemic mistrust and credulity. In contrast, epistemic trust demonstrated a distinct and markedly weaker pattern of associations. These findings should, however, be interpreted with caution, given the limited structural validity and reliability of the underlying measurement model

While our findings originate from a specific population – a largely female sample of university students enrolled in degrees in education – taken together, these results both corroborate and extend prior work, reveal inherent limitations of the German ETMCQ in its initial form, and point to avenues for refinement and future research. The CFAs and reliability analyses underscore the persistent difficulty of reproducing the ETMCQ’s intended three-factor structure in German samples – echoing Weiland et al. [20], who likewise documented misfit and relied on data‐driven adjustments to achieve acceptable fit. The exploratory factor analysis conducted in the present study further highlighted these structural inconsistencies by replicating this instability, showing inconsistent, non‐reproducible item‐to‐factor assignments and especially high overlap between several items as evidenced by substantial cross‐loadings. However, given our study’s highly homogeneous student sample, the results of the EFA should not be construed as a recommendation. Instead, these findings highlight the need for a comprehensive revision and expansion of the ETMCQ item pool and independent, rigorous evaluations of its factorial validity across diverse samples and languages.

Our findings align closely with those of prior psychometric investigations. Campbell et al. [4] reported that the original 18-item English ETMCQ model failed standard CFA cut-offs and required item deletion and the addition of error covariances to yield a 15-item solution. Subsequent translations [26, 28, 29] iterated upon this process, proposing 12- to 14-item variants, yet none achieved uniformly robust fit indices. Weiland et al.’s [20] German adaptation similarly endorsed a 12-item revision with adequate internal consistency (Trust: *ω* = 0.82, *α* = 0.78; Credulity: *ω* = 0.87, *α* = 0.79; Mistrust: *ω* = 0.64, *α* = 0.63), but when applied to our data yielded only marginally acceptable fit. Viewed together, these findings suggest that the psychometric difficulties observed in the German ETMCQ may not merely reflect sample-specific fluctuation, but may point to more general tensions between the intended three-factor conceptualization and its current item-level operationalization across language versions [4, 20, 26, 28, 29]. This has implications not only for the German adaptation, but also for how epistemic trust, mistrust, and credulity are translated into self-report indicators more broadly.

Nomological validity – tested using Weiland et al.’s [20] 12-item model – revealed that epistemic mistrust and credulity both correlated positively with ineffective mentalizing, poorer mental health, and a history of abuse and neglect, and negatively with reappraisal. Credulity alone showed no link to avoidant attachment, whereas both scales were positively associated with anxious attachment, confirming their capture of maladaptive epistemic stances [22, 26, 27]. These patterns are consistent with theoretical accounts that conceptualize epistemic mistrust and credulity as defensive adaptations to relational unpredictability or threat, in which individuals either withdraw from social information (mistrust) or overaccommodate it (credulity) [14]. Moreover, the present associations align with findings from previous studies reporting comparable association patterns [24, 26, 27]. Thus, despite the limited psychometric adequacy of the current German ETMCQ, the pattern of external associations suggests that the comparatively least problematic model still captures some substantively meaningful variance, particularly for epistemic mistrust and credulity. Importantly, however, these theory-consistent associations should not be interpreted as compensating for the limitations in factorial structure and reliability.

In contrast, epistemic trust was unrelated to mentalizing deficits and symptom severity, was only weakly and negatively associated with avoidant attachment and childhood trauma, but showed adaptive links to emotion regulation (positive for reappraisal; negative for suppression). These largely weak and inconsistent associations with key constructs stand in contrast to the central role attributed to epistemic trust within the theoretical model [5, 6, 10, 11], yet correspond to several empirical studies reporting similarly mixed or attenuated patterns of findings [4, 22, 27]. Taken together, this raises questions regarding the current scale’s sensitivity and conceptual breadth, a concern also noted by Weiland et al. [20] and Asgarizadeh et al. [34]. More specifically, the weak associations observed for epistemic trust may reflect limitations in the measurement of this construct rather than its theoretical irrelevance. As highlighted in the present results, the current trust scale shows restricted variability and comparatively low convergent validity, which likely attenuates detectable associations with external criteria.

Importantly, since the completion of the present study, Campbell et al. [57] have published a revised version of the ETMCQ (ETMCQ-R) featuring 18 items with improved factor loadings and model fit. The ETMCQ-R demonstrated enhanced psychometric properties relative to the original scale, particularly regarding internal consistency and structural validity, while maintaining the three-factor structure of trust, mistrust, and credulity. These findings complement our results, which similarly highlight instability in the initial German version of the ETMCQ and underscore the need for a comprehensive revision to achieve adequate psychometric performance. At the same time, they reinforce the broader methodological implication of the present study: psychometric refinement in one language version does not automatically resolve questions of construct representation in another, and therefore cross-linguistic adaptation should be guided not only by fit improvement, but also by careful attention to content coverage, item functioning, and comparability of the underlying constructs.

### Limitations

Several methodological limitations of the current study must be taken into account. First, the cross-sectional design precludes the assessment of temporal stability and test–retest reliability of the ETMCQ scales. Second, all measures relied exclusively on self‐report, which may introduce biases such as social desirability and self‐perception distortions. Participants may have under- or over-reported their epistemic stances to conform to perceived expectations, limiting score accuracy. Third, reliance on a single data collection method raises the risk of shared‐method variance, which can inflate observed associations among constructs. Shared measurement context and response formats may artificially strengthen intercorrelations, complicating the interpretation of the findings. Fourth, because the ETMCQ was administered as part of a broader survey including multiple questionnaires, respondent burden cannot be ruled out entirely. Completing a larger number of self-report measures within a single session may have contributed to fatigue or satisficing and, in turn, may have affected response quality. Future studies may benefit from examining the ETMCQ in shorter or separate assessment settings in order to further rule out possible effects of respondent burden. Fifth, the very nature of the constructs – epistemic trust, epistemic mistrust, and epistemic credulity – challenges the validity of self‐assessment. These dimensions involve subtle social–cognitive processes and unconscious appraisal strategies that individuals may lack the insight or terminology to report accurately. Behavioral paradigms, informant ratings, or implicit assessment techniques may be necessary to capture these constructs comprehensively. Sixth, the gender distribution in our sample was highly unbalanced, with female participants clearly outnumbering male and non-binary participants. This imbalance limits the generalizability of our findings and precluded meaningful gender-specific analyses. Seventh, measurement invariance was not examined in the present study, which limits conclusions regarding subgroup comparability and the broader generalizability of the findings. Finally, and most importantly, the highly homogeneous sample of students enrolled in educational degree programs limits generalizability. Findings may not extend to clinical populations, older adults, or individuals from diverse cultural and educational backgrounds.

### Implications

Considering the limitations of this study, the observed psychometric deficiencies caution against the uncritical use of the current German ETMCQ in research or practice. Researchers should avoid relying solely on the existing item set to operationalize epistemic trust, mistrust, and credulity, given the persistent model misfit, poor convergent validity, and limited composite reliability. Our data indicate that the mistrust and credulity scales may serve as rough proxies for maladaptive epistemic stances, whereas the trust scale currently lacks sufficient psychometric and theoretical support for confident interpretation.

To improve the ETMCQ, we recommend a systematic qualitative pilot – incorporating interviews and expert review – to refine existing items and develop additional ones that comprehensively cover each epistemic dimension. Expanding the item pool to at least four or five items per factor will enhance reliability estimates and facilitate robust AVE indices. In addition, researchers should consider substantially enlarging the item pool – ideally to around ten items per factor – to promote structural stability and broader content coverage. After expanding the item pool, an exploratory factor analysis (EFA) in a large, independent German sample should identify the latent structure, followed by confirmatory factor analyses (CFAs) in separate samples and multi-group CFAs to assess measurement invariance across language versions.

Future validation efforts should employ a multimethod approach to establish convergent and discriminant validity. This could include behavioral paradigms, informant reports, and experimental manipulations of epistemic trust, as well as test–retest studies to determine temporal stability and intervention studies to assess sensitivity to change. Given translation variability, a coordinated evaluation of configural, metric, and scalar invariance across German, English, and other language versions is essential to discern translation artifacts from genuine cultural differences in epistemic stance. Moreover, future research should examine whether the factorial structure and psychometric properties of the ETMCQ and its revised versions differ across gender groups, as gender-related response tendencies may influence the assessment of epistemic stances. Such analyses require sufficiently large and balanced samples and could extend the current state of research on epistemic trust, mistrust, and credulity.

Until a rigorously validated German ETMCQ is available, researchers may consider alternative measures of epistemic stances, such as the Epistemic Trust Assessment [38] or the Epistemic Trust Rating System [58]. While the ETMCQ represents an important advance in self-report measurement of epistemic stances, our findings add to evidence of inconsistent psychometric properties in its current German form. We therefore advocate for a comprehensive revision and multimethod validation of the ETMCQ to realize its potential as a reliable and valid tool for assessing epistemic trust, mistrust, and credulity across diverse populations.

In this regard, the recently published revised version of the ETMCQ [57] represents a promising advancement toward improving the instrument’s psychometric performance and may serve as a valuable reference for future adaptation efforts. However, since the ETMCQ-R is currently available only in English, further cross-linguistic validation and translation work will be necessary before its structure can inform German adaptations.

## Conclusions

In summary, the current German version of the ETMCQ shows limited psychometric adequacy. None of the tested versions demonstrated sufficient reliability or factorial validity. These findings underline the need for a fundamental revision and multimethod validation of the questionnaire before it can be reliably used in research or practice.

## Supplementary Information

Supplementary Material 1.

Supplementary Material 2.

## Data Availability

The data and code used in this study are available upon request. Please contact the first author.

## Abbreviations

AIC: Akaike Information Criterion
AVE: Average variance extracted
BIC: Bayesian Information Criterion
CFA: Confirmatory Factor Analysis
CFI: Comparative Fit Index
CTQ: Childhood Trauma Questionnaire
ECR: RD8–Experiences in Close Relationships–Revised, Short Form (8 items)
EFA: Exploratory Factor Analysis
ERQ: Emotion Regulation Questionnaire
ETMCQ: Epistemic Trust, Mistrust, and Credulity Questionnaire
ETMCQ: R–Epistemic Trust, Mistrust, and Credulity Questionnaire Revised
KMO: Kaiser–Meyer–Olkin Measure of Sampling Adequacy
RFQ: Reflective Functioning Questionnaire
RMSEA: Root Mean Square Error of Approximation
SCL: Symptom Checklist
SRMR: Standardized Root Mean Square Residual
TLI: Tucker–Lewis Index

## References

[1] Fricker M. Epistemic injustice: power and ethics of knowing. Oxford: Oxford University Press; 2007.

[2] Beck U, Giddens A, Lash S. Reflexive modernization: politics, tradition and aesthetics in the modern social order. Stanford: Stanford University Press; 1994.

[3] Origgi G. Epistemic injustice and epistemic trust. Soc Epistemol. 2012;26(2):221–35. 10.1080/02691728.2011.652213.

[4] Campbell C, Tanzer M, Saunders R, et al. Development and validation of a self-report measure of epistemic trust. PLoS ONE. 2021;16(4):e0250264. 10.1371/journal.pone.0250264. 33861805 10.1371/journal.pone.0250264 PMC8051785

[5] Fonagy P, Allison E. The role of mentalizing and epistemic trust in the therapeutic relationship. Psychotherapy. 2014;51(3):372–80. 10.1037/a0036505. 24773092 10.1037/a0036505

[6] Luyten P, Campbell C, Allison E, Fonagy P. The mentalizing approach to psychopathology: state of the art and future directions. Annu Rev Clin Psychol. 2020;16:297–325. 10.1146/annurev-clinpsy-071919-015355. 32023093 10.1146/annurev-clinpsy-071919-015355

[7] Sperber D, Clément F, Heintz C, et al. Epistemic vigilance. Mind Lang. 2010;25(4):359–93. 10.1111/j.1468-0017.2010.01394.x.

[8] Wilson DB, Sperber D. Meaning and relevance. Cambridge: Cambridge University Press; 2012.

[9] Csibra G, Gergely G. Natural pedagogy. Trends Cogn Sci. 2009;13(4):148–53. 10.1016/j.tics.2009.01.005. 19285912 10.1016/j.tics.2009.01.005

[10] Fonagy P, Luyten P, Allison E. Epistemic petrification and the restoration of epistemic trust: a new conceptualization of borderline personality disorder and its psychosocial treatment. J Pers Disord. 2015;29(5):575–609. 10.1521/pedi.2015.29.5.575. 26393477 10.1521/pedi.2015.29.5.575

[11] Fonagy P, Luyten P, Allison E, Campbell C. Mentalizing, epistemic trust and the phenomenology of psychotherapy. Psychopathology. 2019;52(2):94–103. 10.1159/000501526. 31362289 10.1159/000501526

[12] Fonagy P, Campbell C, Constantinou M, et al. Culture and psychopathology: an attempt at reconsidering the role of social learning. Dev Psychopathol. 2022;34(4):1205–20. 10.1017/S0954579421000092. 33766162 10.1017/S0954579421000092

[13] Corriveau KH, Harris PL, Meins E, et al. Young children’s trust in their mother’s claims: longitudinal links with attachment security in infancy. Child Dev. 2009;80(3):750–61. 10.1111/j.1467-8624.2009.01295.x. 19489901 10.1111/j.1467-8624.2009.01295.x

[14] Fonagy P, Luyten P, Allison E, Campbell C. What we have changed our minds about: Part 1. Borderline personality disorder as a limitation of resilience. Borderline Personal Disord Emot Dysregul. 2017;4:11. 10.1186/s40479-017-0061-9. 28413687 10.1186/s40479-017-0061-9 PMC5389119

[15] Li E, Campbell C, Midgley N, Luyten P. Epistemic trust: a comprehensive review of empirical insights and implications for developmental psychopathology. Res Psychother. 2023;26(3):704. 10.4081/ripppo.2023.704. 38156560 10.4081/ripppo.2023.704 PMC10772859

[16] Knapen S, Hutsebaut J, van Diemen R, Beekman A. Epistemic trust as a psycho-marker for outcome in psychosocial interventions. J Infant Child Adolesc Psychother. 2020;19(4):417–26. 10.1080/15289168.2020.1812322.

[17] Nolte T, Hutsebaut J, Sharp C, et al. The role of epistemic trust in mentalization-based treatment of borderline psychopathology. J Pers Disord. 2023;37(5):633–59. 10.1521/pedi.2023.37.5.633.

[18] Fonagy P, Luyten P, Allison E, Campbell C. What we have changed our minds about: Part 2. Borderline personality disorder, epistemic trust and the developmental significance of social communication. Borderline Personal Disord Emot Dysregul. 2017;4:9. 10.1186/s40479-017-0062-8. 28405338 10.1186/s40479-017-0062-8 PMC5387344

[19] Campbell C, Allison E. Mentalizing the modern world. Psychoanal Psychother. 2022;36(3):206–17. 10.1080/02668734.2022.2089906.

[20] Weiland AM, Taubner S, Zettl M, et al. Epistemic trust and associations with psychopathology: validation of the German version of the Epistemic Trust, Mistrust and Credulity-Questionnaire (ETMCQ). PLoS ONE. 2024;19(11):e0312995. 10.1371/journal.pone.0312995. 39541339 10.1371/journal.pone.0312995 PMC11563411

[21] Knapen SRY, Mensink W, Hoogendoorn AW, et al. Associations between childhood trauma and epistemic trust, attachment, mentalizing, and symptoms of borderline personality disorder. Psychopathology. 2025. 10.1159/000542919. 39809247 10.1159/000542919

[22] Kampling H, Riedl D, Lampe A, et al. Somatic symptom disorder and the role of epistemic trust, personality functioning and child abuse: Results from a population-based representative German sample. J Affect Disord. 2025;373:429–37. 10.1016/j.jad.2024.12.096. 39736403 10.1016/j.jad.2024.12.096

[23] Kumpasoğlu GB, Saunders R, Campbell C, et al. Mentalizing, epistemic trust and interpersonal problems in emotion regulation: a sequential path analysis across common mental health disorders and community control samples. J Affect Disord. 2025;372:502–11. 10.1016/j.jad.2024.12.050. 39694336 10.1016/j.jad.2024.12.050

[24] Tironi M, Charpentier Mora S, Liotti M, et al. Adverse childhood experiences and psychological maladjustment in adolescence: the protective role of epistemic trust, mentalized affectivity, and reflective functioning. J Clin Psychol. 2024;80(11):2228–46. 10.1002/jclp.23733. 39101491 10.1002/jclp.23733

[25] Kampling H, Kruse J, Lampe A, et al. Epistemic trust and personality functioning mediate the association between adverse childhood experiences and posttraumatic stress disorder and complex posttraumatic stress disorder in adulthood. Front Psychiatry. 2022;13:919191. 10.3389/fpsyt.2022.919191. 36032256 10.3389/fpsyt.2022.919191 PMC9399466

[26] Liotti M, Milesi A, Spitoni GF, et al. Unpacking trust: the Italian validation of the Epistemic Trust, Mistrust, and Credulity Questionnaire (ETMCQ). PLoS ONE. 2023;18(1):e0280328. 10.1371/journal.pone.0280328. 36701301 10.1371/journal.pone.0280328 PMC9879475

[27] Bincoletto A, Liotti M, Di Giuseppe M, et al. The interplay of epistemic trust, defensive mechanisms, interpersonal problems, and symptomatology: an empirical investigation. Pers Individ Dif. 2025;233:112893. 10.1016/j.paid.2024.112893.

[28] Greiner C, Besch V, Bouchard-Boivin M, et al. Epistemic trust, mistrust and credulity questionnaire (ETMCQ) validation in French language: exploring links to loneliness. PLoS ONE. 2025;20(3):e0303918. 10.1371/journal.pone.0303918. 40117268 10.1371/journal.pone.0303918 PMC11927912

[29] Asgarizadeh A, Ghanbari S. Iranian adaptation of the ETMCQ: Iranian adaptation of the Epistemic Trust, Mistrust, and Credulity Questionnaire (ETMCQ): validity, reliability, discriminant ability, and sex invariance. Brain Behav. 2024;14(3):e3455. 10.1002/brb3.3455. 38451001 10.1002/brb3.3455 PMC10918607

[30] Brauner F, Fonagy P, Campbell C, et al. Trust me, do not trust anyone: how epistemic mistrust and credulity are associated with conspiracy mentality. Res Psychother. 2023;26(3):705. 10.4081/ripppo.2023.705. 38156557 10.4081/ripppo.2023.705 PMC10782893

[31] Hauschild S, Kasper LA, Berning A, Taubner S. The relationship between epistemic stance, mentalizing, paranoid distress and conspiracy mentality. Res Psychother. 2023;26(3):706. 10.4081/ripppo.2023.706. 38156598 10.4081/ripppo.2023.706 PMC10782896

[32] Orme W, Bowersox L, Vanwoerden S, et al. The relation between epistemic trust and borderline pathology in an adolescent inpatient sample. Borderline Personal Disord Emot Dysregul. 2019;6:13. 10.1186/s40479-019-0110-7. 31485332 10.1186/s40479-019-0110-7 PMC6712815

[33] Locati F, Benzi IMA, Milesi A, et al. Associations of mentalization and epistemic trust with internalizing and externalizing problems in adolescence: A gender-sensitive structural equation modeling approach. J Adolesc. 2023;95(8):1564–77. 10.1002/jad.12226. 37500187 10.1002/jad.12226

[34] Asgarizadeh A, Hunjani M, Ghanbari S. Do we really need trust? the incremental validity of epistemic trust over epistemic mistrust and credulity in predicting mentalizing-related constructs? Asian J Psychiatry. 2023;87:103688. 10.1016/j.ajp.2023.103688. 10.1016/j.ajp.2023.103688 37413922

[35] Riedl D, Kampling H, Kruse J, et al. Epistemic trust as a critical success factor in psychosomatic rehabilitation – results from a naturalistic multi-center observational study. J Clin Med. 2023;13(1):177. 10.3390/jcm13010177. 38202184 10.3390/jcm13010177 PMC10780285

[36] Riedl D, Rothmund MS, Grote V, et al. Mentalizing and epistemic trust as critical success factors in psychosomatic rehabilitation: results of a single center longitudinal observational study. Front Psychiatry. 2023;14:1150422. 10.3389/fpsyt.2023.1150422. 37252135 10.3389/fpsyt.2023.1150422 PMC10213326

[37] Schwarzer NH, Behringer N, Dees P, et al. Effectiveness of mentalization-based training for teachers and educators: results of a pilot study. Prävention & Gesundheitsförderung; 2025. 10.1007/s11553-025-01209-4.

[38] Schröder-Pfeifer P, Georg AK, Talia A, et al. The epistemic trust assessment—an experimental measure of epistemic trust. Psychoanal Psychol. 2022;39(1):50–8. 10.1037/pap0000322.

[39] Knapen S, Swildens WE, Mensink W, et al. The development and psychometric evaluation of the Questionnaire Epistemic Trust (QET): a self-report assessment of epistemic trust. Clin Psychol Psychother. 2023. 10.1002/cpp.2930. 37947067 10.1002/cpp.2930

[40] World Medical Association. World medical association declaration of helsinki: ethical principles for medical research involving human subjects. JAMA. 2024;331(2):219–25. 10.1001/jama.2024.21972. 10.1001/jama.2024.21972 39425955

[41] Bernstein DP, Stein JA, Newcomb MD, et al. Development and validation of a brief screening version of the Childhood Trauma Questionnaire. Child Abuse Negl. 2003;27(2):169–90. 10.1016/s0145-2134(02)00541-0. 12615092 10.1016/s0145-2134(02)00541-0

[42] Wingenfeld K, Spitzer C, Mensebach C, et al. The German version of the Childhood Trauma Questionnaire (CTQ): preliminary psychometric properties. Psychother Psychosom Med Psychol. 2010;60(11):442–50. 10.1055/s-0030-1247564. 20200804 10.1055/s-0030-1247564

[43] Fonagy P, Luyten P, Moulton-Perkins A, et al. Development and validation of a self-report measure of mentalizing: the reflective functioning questionnaire. PLoS ONE. 2016;11(7):e0158678. 10.1371/journal.pone.0158678. 27392018 10.1371/journal.pone.0158678 PMC4938585

[44] Müller S, Wendt LP, Spitzer C, et al. A critical evaluation of the reflective functioning questionnaire. J Pers Assess. 2022;104(5):613–27. 10.1080/00223891.2021.1981346. 34597256 10.1080/00223891.2021.1981346

[45] Ehrenthal JC, Zimmermann J, Brenk-Franz K, et al. Short version of the ECR-RD8. BMC Psychol. 2021;9(1):140. 10.1186/s40359-021-00637-z. 34521473 10.1186/s40359-021-00637-z PMC8439023

[46] Gross JJ, John OP. Individual differences in two emotion regulation processes: implications for affect, relationships, and well-being. J Pers Soc Psychol. 2003;85(2):348–62. 10.1037/0022-3514.85.2.348. 12916575 10.1037/0022-3514.85.2.348

[47] Abler B, Kessler H. Emotion regulation questionnaire—a German version of the ERQ by Gross and John. Diagnostica. 2009;55:144–52. 10.1026/0012-1924.55.3.144.

[48] Derogatis LR. SCL-90-R manual. Minneapolis, MN: National Computer Systems; 1994.

[49] Petrowski K, Schmalbach B, Kliem S, et al. Symptom-Checklist-K-9: norm values and factorial structure in a representative German sample. PLoS ONE. 2019;14(4):e0213490. 10.1371/journal.pone.0213490. 30951538 10.1371/journal.pone.0213490 PMC6450622

[50] Wickham H, Miller E, Smith D. haven: Import and export ‘SPSS’, ‘Stata’ and ‘SAS’ files. R package version 2 5 5. 2025. 10.32614/CRAN.package.haven.

[51] Rosseel Y, Jorgensen TD, De Wilde L, lavaan. Latent variable analysis. R package version 0.6–20. 2025. 10.32614/CRAN.package.lavaan.

[52] Wickham H, François R, Henry L, Müller K, Vaughan D. dplyr: a grammar of data manipulation. R package version 1 1 4. 2023. 10.32614/CRAN.package.dplyr.

[53] Hu LT, Bentler PM. Cutoff criteria for fit indexes in covariance structure analysis: conventional criteria versus new alternatives. Struct Equ Model. 1999;6(1):1–55. 10.1080/10705519909540118.

[54] Kline RB. Principles and practice of structural equation modeling. 4th ed. New York: Guildford; 2016.

[55] Fornell C, Larcker DF. Evaluating structural equation models with unobservable variables and measurement error. J Mark Res. 1981;18(1):39–50. 10.2307/3151312.

[56] Hair JF, Black WC, Babin BJ, Anderson RE. Multivariate data analysis. 7th Edition. Pearson Education; 2014. https://elibrary.pearson.de/book/99.150005/9781292035116?lang=de

[57] Campbell C, Delamain H, Saunders R, et al. Development and validation of the Revised Epistemic Trust, Mistrust and Credulity Questionnaire (ETMCQ-R). BJPsych open. 2025;11(5):e191. 10.1192/bjo.2025.10813. 40888372 10.1192/bjo.2025.10813 PMC12451556

[58] Fisher S, Guralnik T, Fonagy P, Zilcha-Mano S. The development of the Epistemic Trust Rating System (ETRS). Psychother Res. 2024;35(3):412–23. 10.1080/10503307.2023.2299213. 38185139 10.1080/10503307.2023.2299213

