# Supplementary material for: Replicability or revision? Further evidence on the psychometric quality of the German version of the epistemic trust, mistrust and credulity questionnaire
Source: BMC Psychol. 2026 May 26;14:781. doi: 10.1186/s40359-026-04810-0 (PMC13202747; doi:10.1186/s40359-026-04810-0)
Supplement: Supplementary file 1 — Supplementary Material 1. [file 40359_2026_4810_MOESM1_ESM.docx]

**Electronic Supplementary Material 1**

*Replicability or revision? Further evidence on the psychometric quality of the German version of the Epistemic Trust, Mistrust and Credulity Questionnaire*

Supplementary materials prepared for the revised manuscript

This supplementary file provides **Supplementary Table S1**, which documents the item composition of all three ETMCQ model specifications including the full German item wording, as well as **Supplementary Figures S1–S3**, which show the corresponding path diagrams.

**Supplementary Table S1. Item composition of the three ETMCQ models**

| **Item** | **German item wording** | **Theoretical factor** | **Unmodified model** | **Campbell et al. model** | **Weiland et al. model** |
| --- | --- | --- | --- | --- | --- |
| ETMCQ1 | Ich frage üblicherweise andere um Rat, wenn ich persönliche Probleme habe. | Trust | X | X | X |
| ETMCQ2 | Ich finde es leichter, Informationen zu vertrauen und aufzunehmen, wenn sie von jemandem stammen, der mich gut kennt. | Trust | X | X | X |
| ETMCQ3 | Ich bevorzuge, Dinge im Internet selbst herauszufinden, statt andere um Informationen zu bitten. | Mistrust | X | X | – |
| ETMCQ4 | Ich habe oft das Gefühl, dass andere nicht verstehen, was ich will und brauche. | Mistrust | X | X | X |
| ETMCQ5 | Ich werde oft für naiv gehalten, weil ich fast alles glaube, was andere mir erzählen. | Credulity | X | X | X |
| ETMCQ6 | Wenn ich mit verschiedenen Menschen spreche, kann ich mich leicht von dem überzeugen lassen, was sie sagen, auch wenn dies etwas anders ist, als das was ich vorher geglaubt habe. | Credulity | X | X | – |
| ETMCQ7 | Ein Gespräch mit Menschen, die mich schon lange kennen, kann mir helfen, neue Perspektiven über mich selbst zu entwickeln. | Trust | X | X | X |
| ETMCQ8 | Ich finde es sehr nützlich, aus dem zu lernen, was andere mir über ihre Erfahrungen erzählen. | Trust | X | X | X |
| ETMCQ9 | Wenn du dem, was andere dir erzählen, zu viel Glauben schenkst, bist du leichter verletzbar. | Mistrust | X | X | X |
| ETMCQ10 | Wenn mir jemand etwas erzählt, frage ich mich sofort, warum er mir das jetzt erzählt. | Mistrust | X | X | X |
| ETMCQ11 | Ich habe zu oft Ratschläge von den falschen Menschen angenommen. | Credulity | X | X | X |
| ETMCQ12 | Verschiedene Leute haben mir gesagt, dass ich zu leicht von anderen beeinflussbar bin. | Credulity | X | X | X |
| ETMCQ13 | Wenn ich nicht weiß, was ich tun soll, ist mein erster Impuls, jemanden zu fragen, dessen Meinung ich schätze. | Trust | X | X | X |
| ETMCQ14 | Ich befolge normalerweise keinen Ratschlag, den ich von anderen bekomme, selbst wenn ich denke, dass der Rat wahrscheinlich gut ist. | Mistrust | X | X | – |
| ETMCQ15 | In der Vergangenheit habe ich falsch eingeschätzt, wem ich glauben kann, und bin deswegen ausgenutzt worden. | Credulity | X | X | X |

**Note.** The unmodified model included all 15 items without correlated residuals. The Campbell et al. model retained the same 15 items but additionally specified correlated residuals between ETMCQ7 and ETMCQ8, ETMCQ5 and ETMCQ6, ETMCQ5 and ETMCQ12, and ETMCQ6 and ETMCQ12. The Weiland et al. model retained 12 items and additionally specified correlated residuals between ETMCQ7 and ETMCQ8 and between ETMCQ5 and ETMCQ12.

**Supplementary Figure S1. Path diagram of the unmodified 15-item ETMCQ model**


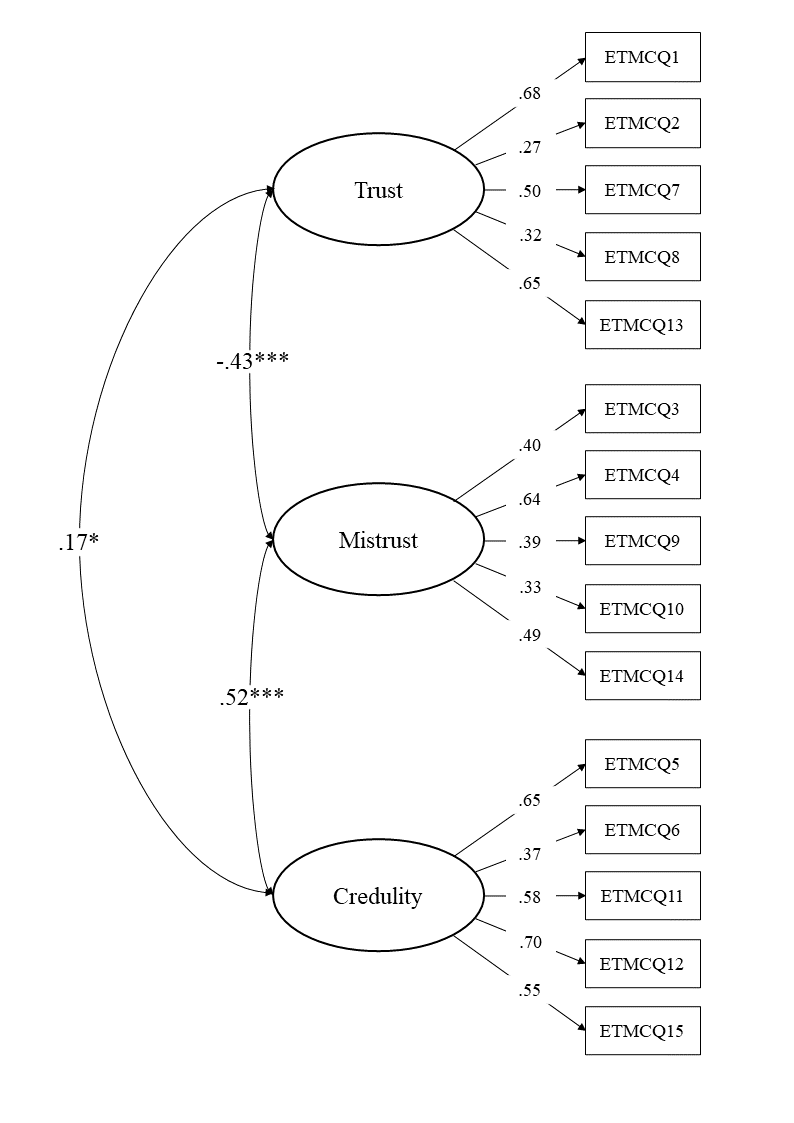


**Note.** Standardized path diagram of the unmodified three-factor ETMCQ model including the trust, mistrust, and credulity factors and their respective item assignments.

**Supplementary Figure S2. Path diagram of the Campbell et al. ETMCQ model**


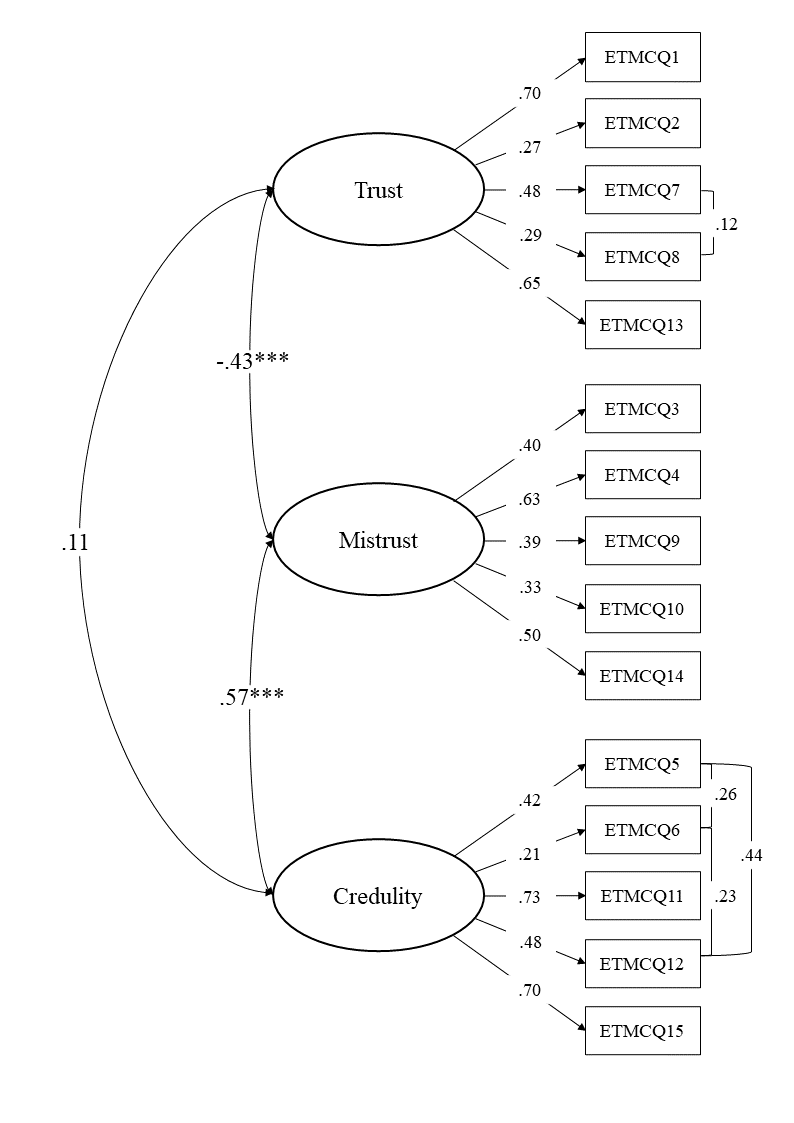


**Note.** Standardized path diagram of the modified 15-item ETMCQ model including the specified correlated residuals between ETMCQ7 and ETMCQ8, ETMCQ5 and ETMCQ6, ETMCQ5 and ETMCQ12, and ETMCQ6 and ETMCQ12.

**Supplementary Figure S3. Path diagram of the Weiland et al. ETMCQ model**


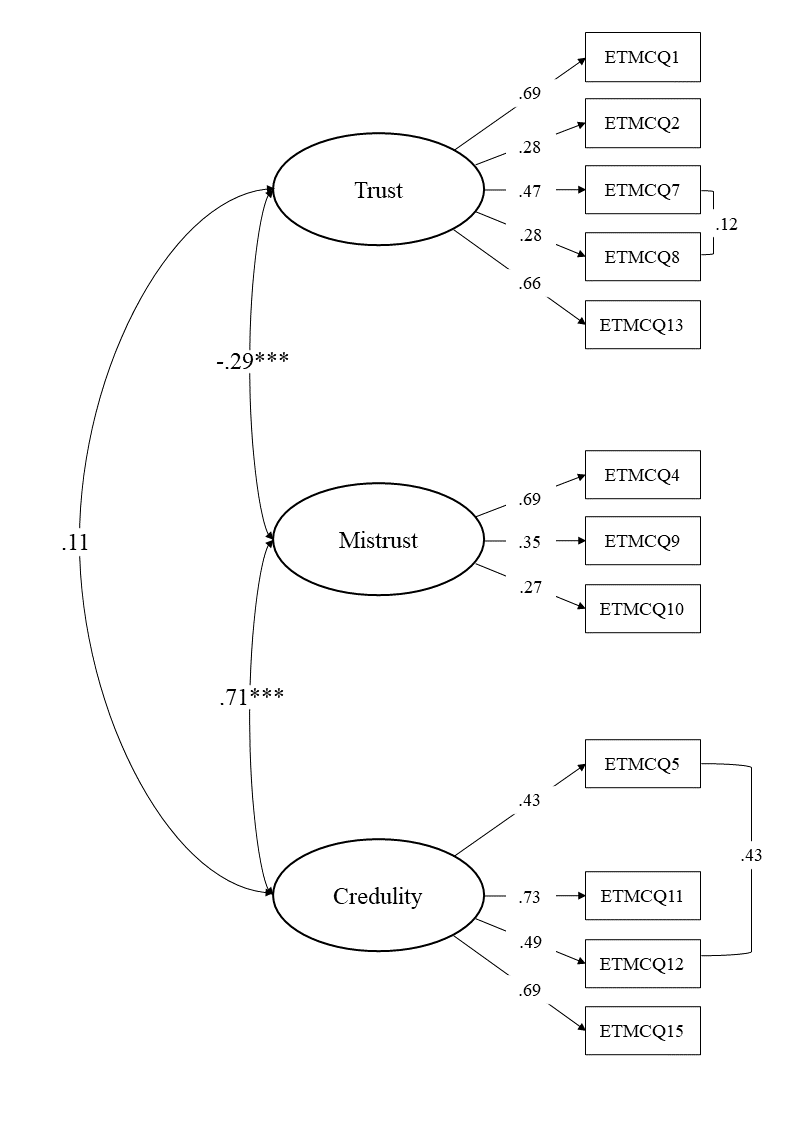


**Note.** Standardized path diagram of the 12-item German ETMCQ model including the specified correlated residuals between ETMCQ7 and ETMCQ8 and ETMCQ5 and ETMCQ12.
