# Supplementary material for: Replicability or revision? Further evidence on the psychometric quality of the German version of the epistemic trust, mistrust and credulity questionnaire
Source: BMC Psychol. 2026 May 26;14:781. doi: 10.1186/s40359-026-04810-0 (PMC13202747; doi:10.1186/s40359-026-04810-0)
Supplement: Supplementary file 2 — Supplementary Material 2. [file 40359_2026_4810_MOESM2_ESM.docx]

**Electronic Supplementary Material**

*Replicability or revision? Further evidence on the psychometric quality of the German version of the Epistemic Trust, Mistrust and Credulity Questionnaire*

**Supplementary Table S2. Item-level descriptive statistics for the German ETMCQ**

| **Item** | **M** | **SD** | **Skewness** | **Kurtosis** |
| --- | --- | --- | --- | --- |
| ETMCQ_1 | 5.199 | 1.445 | -0.872 | 0.050 |
| ETMCQ_2 | 5.594 | 1.150 | -1.281 | 2.224 |
| ETMCQ_3 | 3.765 | 1.561 | 0.025 | -0.893 |
| ETMCQ_4 | 3.446 | 1.631 | 0.292 | -1.003 |
| ETMCQ_5 | 2.808 | 1.687 | 0.698 | -0.634 |
| ETMCQ_6 | 3.670 | 1.469 | 0.092 | -0.872 |
| ETMCQ_7 | 6.007 | 0.932 | -1.228 | 2.303 |
| ETMCQ_8 | 5.772 | 1.019 | -1.116 | 2.131 |
| ETMCQ_9 | 3.744 | 1.591 | 0.020 | -1.022 |
| ETMCQ_10 | 3.266 | 1.600 | 0.275 | -1.069 |
| ETMCQ_11 | 2.319 | 1.364 | 1.217 | 0.893 |
| ETMCQ_12 | 2.644 | 1.620 | 0.772 | -0.596 |
| ETMCQ_13 | 5.653 | 1.362 | -1.165 | 0.918 |
| ETMCQ_14 | 2.145 | 1.055 | 1.277 | 2.086 |
| ETMCQ_15 | 3.180 | 1.855 | 0.538 | -0.976 |

Note. N = 567. M = mean; SD = standard deviation.

**Supplementary Table S3. Inter-item correlations of the German ETMCQ items**

| **Item** | **1** | **2** | **3** | **4** | **5** | **6** | **7** | **8** | **9** | **10** | **11** | **12** | **13** | **14** | **15** |
| --- | --- | --- | --- | --- | --- | --- | --- | --- | --- | --- | --- | --- | --- | --- | --- |
| ETMCQ_1 | 1.000 | 0.139 | -0.249 | -0.170 | 0.079 | 0.112 | 0.320 | 0.181 | -0.070 | -0.109 | 0.057 | 0.049 | 0.468 | -0.195 | 0.008 |
| ETMCQ_2 | 0.139 | 1.000 | -0.033 | 0.007 | 0.063 | 0.071 | 0.235 | 0.082 | 0.098 | 0.103 | 0.053 | 0.043 | 0.223 | -0.024 | 0.001 |
| ETMCQ_3 | -0.249 | -0.033 | 1.000 | 0.199 | -0.006 | 0.045 | -0.192 | -0.098 | 0.218 | 0.164 | 0.032 | 0.025 | -0.198 | 0.235 | 0.099 |
| ETMCQ_4 | -0.170 | 0.007 | 0.199 | 1.000 | 0.244 | 0.126 | -0.125 | -0.124 | 0.216 | 0.187 | 0.344 | 0.301 | -0.106 | 0.297 | 0.314 |
| ETMCQ_5 | 0.079 | 0.063 | -0.006 | 0.244 | 1.000 | 0.315 | 0.056 | 0.072 | 0.115 | -0.007 | 0.267 | 0.550 | 0.157 | 0.065 | 0.312 |
| ETMCQ_6 | 0.112 | 0.071 | 0.045 | 0.126 | 0.315 | 1.000 | 0.056 | 0.127 | 0.018 | 0.024 | 0.172 | 0.297 | 0.106 | -0.033 | 0.107 |
| ETMCQ_7 | 0.320 | 0.235 | -0.192 | -0.125 | 0.056 | 0.056 | 1.000 | 0.234 | -0.066 | -0.096 | 0.000 | -0.028 | 0.282 | -0.179 | 0.072 |
| ETMCQ_8 | 0.181 | 0.082 | -0.098 | -0.124 | 0.072 | 0.127 | 0.234 | 1.000 | -0.044 | 0.011 | -0.046 | -0.043 | 0.190 | -0.208 | -0.034 |
| ETMCQ_9 | -0.070 | 0.098 | 0.218 | 0.216 | 0.115 | 0.018 | -0.066 | -0.044 | 1.000 | 0.182 | 0.148 | 0.189 | 0.017 | 0.203 | 0.231 |
| ETMCQ_10 | -0.109 | 0.103 | 0.164 | 0.187 | -0.007 | 0.024 | -0.096 | 0.011 | 0.182 | 1.000 | 0.140 | 0.098 | -0.027 | 0.235 | 0.047 |
| ETMCQ_11 | 0.057 | 0.053 | 0.032 | 0.344 | 0.267 | 0.172 | 0.000 | -0.046 | 0.148 | 0.140 | 1.000 | 0.354 | 0.055 | 0.142 | 0.523 |
| ETMCQ_12 | 0.049 | 0.043 | 0.025 | 0.301 | 0.550 | 0.297 | -0.028 | -0.043 | 0.189 | 0.098 | 0.354 | 1.000 | 0.118 | 0.078 | 0.299 |
| ETMCQ_13 | 0.468 | 0.223 | -0.198 | -0.106 | 0.157 | 0.106 | 0.282 | 0.190 | 0.017 | -0.027 | 0.055 | 0.118 | 1.000 | -0.155 | 0.046 |
| ETMCQ_14 | -0.195 | -0.024 | 0.235 | 0.297 | 0.065 | -0.033 | -0.179 | -0.208 | 0.203 | 0.235 | 0.142 | 0.078 | -0.155 | 1.000 | 0.136 |
| ETMCQ_15 | 0.008 | 0.001 | 0.099 | 0.314 | 0.312 | 0.107 | 0.072 | -0.034 | 0.231 | 0.047 | 0.523 | 0.299 | 0.046 | 0.136 | 1.000 |

Note. N = 567. Correlations are Pearson correlations.

**Supplementary Table S4. Item-level diagnostics for the German ETMCQ**

| **Scale** | **Item** | **Corrected item-total correlation** | **Cronbach's alpha if item deleted** | **McDonald's omega if item deleted** |
| --- | --- | --- | --- | --- |
| Trust | ETMCQ_1 | 0.448 | 0.503 | 0.520 |
| Trust | ETMCQ_2 | 0.246 | 0.609 | 0.621 |
| Trust | ETMCQ_7 | 0.417 | 0.537 | 0.559 |
| Trust | ETMCQ_8 | 0.249 | 0.604 | 0.620 |
| Trust | ETMCQ_13 | 0.482 | 0.481 | 0.518 |
| Mistrust | ETMCQ_3 | 0.311 | 0.512 | 0.534 |
| Mistrust | ETMCQ_4 | 0.342 | 0.493 | 0.511 |
| Mistrust | ETMCQ_9 | 0.318 | 0.508 | 0.535 |
| Mistrust | ETMCQ_10 | 0.289 | 0.526 | 0.543 |
| Mistrust | ETMCQ_14 | 0.386 | 0.491 | 0.492 |
| Credulity | ETMCQ_5 | 0.530 | 0.617 | 0.642 |
| Credulity | ETMCQ_6 | 0.301 | 0.708 | 0.714 |
| Credulity | ETMCQ_11 | 0.486 | 0.643 | 0.669 |
| Credulity | ETMCQ_12 | 0.552 | 0.609 | 0.628 |
| Credulity | ETMCQ_15 | 0.432 | 0.666 | 0.678 |

Note. Corrected item-total correlations as well as Cronbach's alpha and McDonald's omega if item deleted were calculated within the theoretically assigned ETMCQ subscales.

**Supplementary Table S5. Subscale reliability coefficients of the German ETMCQ**

| **Scale** | **n items** | **Cronbach's alpha** | **McDonald's omega** |
| --- | --- | --- | --- |
| Trust | 5 | 0.607 | 0.620 |
| Mistrust | 5 | 0.561 | 0.578 |
| Credulity | 5 | 0.700 | 0.709 |

Note. Reliability coefficients are reported for the original three ETMCQ subscales.
